# Hesperetin Exhibits Anti-Inflammatory Effects on Chondrocytes via the AMPK Pathway to Attenuate Anterior Cruciate Ligament Transection-Induced Osteoarthritis

**DOI:** 10.3389/fphar.2021.735087

**Published:** 2021-09-16

**Authors:** Jiaqin Wu, Yuna Qian, Cheng Chen, Fan Feng, Lianhong Pan, Li Yang, Chunli Wang

**Affiliations:** ^1^ National Innovation and Attracting Talents “111” Base, Key Laboratory of Biorheological Science and Technology, Ministry of Education, College of Bioengineering, Chongqing University, Chongqing, China; ^2^ Engineering Research Center of Clinical Functional Materials and Diagnosis and Treatment Devices of Zhejiang Province, Wenzhou Institute, University of Chinese Academy of Sciences, Wenzhou, China; ^3^ Department of Orthopaedics, The First Affiliated Hospital of Chongqing Medical University, Chongqing, China

**Keywords:** osteoarthritis, inflammation, cartilage degeneration, hesperetin, AMPK

## Abstract

This study aimed to determine whether hesperetin (HPT) has chondroprotective effects against the TNF-α-induced inflammatory response of chondrocytes and related mechanisms and clarify the impact of HPT on osteoarthritis (OA) induced by anterior cruciate ligament transection (ACLT). Under tumor necrosis factor-α (TNF-α) stimulation, rat chondrocytes were treated with or without HPT. The CCK-8 assay was used to detect viability and cytotoxicity. RT-qPCR and Western blot were used to examine the expression of aggrecan, collagen type II, and inflammatory and proliferative genes/proteins in chondrocytes. Flow cytometry was used to check the cell cycle to determine whether HPT protects chondrocytes against the inhibitory effect of TNF-α on chondrocyte proliferation. In addition, RNA sequencing was used to discover possible molecular targets and pathways and then validate these pathways with specific protein phosphorylation levels. Finally, immunofluorescence staining was used to examine the phosphorylation of the AMP-activated protein kinase (AMPK) pathway. The results showed that HPT restored the upregulation of interleukin 1β (IL-1β), PTGS2, and MMP-13 induced by TNF-α. In addition, HPT reversed the degradation of the extracellular matrix of chondrocytes induced by TNF-α. HPT also reversed the inhibitory effect of TNF-α on chondrocyte proliferation. RNA sequencing revealed 549 differentially expressed genes (DEGs), of which 105 were upregulated and 444 were downregulated, suggesting the potential importance of the AMPK pathway. Progressive analysis showed that HPT mediated the repair of TNF-α-induced chondrocyte damage through the AMPK signaling pathway. Thus, local treatment of HPT can improve OA induced by ACLT. These findings indicated that HPT has significant protective and anti-inflammatory effects on chondrocytes through the AMPK signaling pathway, effectively preventing cartilage degradation. Given the various beneficial effects of HPT, it can be used as a potential natural drug to treat OA.

## Introduction

Osteoarthritis (OA) is a connective tissue degenerative disease and frequently associated with joint inflammation and substantial pain, possibly debilitating the affected patient (Dieppe and Lohmander, 2005; Neogi, 2013; Glyn-Jones et al., 2015; Lin et al., 2016; Wang et al., 2020a). It is characterized by the loss of the articular cartilage and subchondral bone reactive hyperplasia due to repeated inflammatory responses (Chen et al., 2017). Growing evidence indicates that the inflammatory response induced by elevated levels of proinflammatory cytokines, including tumor necrosis factor- (TNF-) α and interleukin- (IL-) 1β, plays a critical role in the pathogenesis of OA (Robinson et al., 2016; Wang and He, 2018). Although osteoarthritis is an epidemic, the drugs for this condition are far from ideal (Wang et al., 2015; Zhu et al., 2018). Currently available therapeutic strategies for OA include injectable glucocorticoid compounds and nonsteroidal anti-inflammatory drugs (NSAIDs) (Deyle et al., 2020; Kolasinski et al., 2020). However, these drugs only offer symptomatic relief, with no effect on disease modification or progression. Therefore, it is imperative to seek effective medicines to prevent OA.

TNF-α is a key inflammatory factor, with a leading role in the inflammatory process. Several studies have demonstrated that TNF-α downregulates the synthesis of major extracellular matrix (ECM) components by inhibiting the anabolic activities of chondrocytes (Saklatvala, 1986). It has been reported that inflammatory cytokines exert a destructive effect on cartilage by inhibiting the anabolic activities of chondrocytes (Zhao et al., 2015) and activated chondrocytes to produce matrix metalloproteinases (MMPs), especially MMP-13 (a key regulator of cartilage destruction) (Gonzalez-Rey et al., 2006). TNF-α activates the NF-κB signaling pathway in various medical conditions, which in turn further facilitates the proinflammatory function of TNF-α (Hayden and Ghosh, 2014). Apart from their destructive effects, inflammatory cytokines also tend to induce apoptosis in chondrocytes (Hosseinzadeh et al., 2016). Moreover, recent data suggest that TNF-α is involved in cartilage degeneration in OA (Hosseinzadeh et al., 2016).

With rapid advances in natural products research, active ingredients in natural products have demonstrated their therapeutic effects, with fewer side effects in clinical practice. Hesperetin (HPT), a natural flavonoid from the *Citrus L.* plant in the *Rutaceae* family, has diverse pharmacological capacities, including anti-inflammatory and antioxidative properties (Parhiz et al., 2015). HPT has widely been used to prevent and treat various chronic inflammatory conditions (Chen et al., 2019; Muhammad et al., 2019; Wang et al., 2020c). Although HPT is known to significantly inhibit IL-1β-induced inflammatory responses in human chondrocytes during osteoarthritis (Lin et al., 2020), it remains unknown whether exogenously applied HPT is capable of reversing TNF-α-induced abnormal expression of genes and proteins to prevent the degradation of articular cartilages induced by ACLT transection injury. Moreover, the specific molecular mechanism of this effect is still unknown.

This study investigated whether HPT has chondroprotective effects against the inflammatory responses of chondrocytes induced by TNF-α and the related mechanisms. First, an *in vitro* OA model of chondrocytes (with TNF-α induction) was prepared to assess the inhibitory effects of HPT on the inflammation caused by abnormal gene/protein phenotypic changes. To further unravel the underlying molecular mechanisms, high throughput RNA sequencing quantification was used to analyze mRNA-level changes induced by TNF-α with or without HPT treatment in global chondrocytes. Finally, the AMPK signaling pathway was demonstrated to relieve OA, which could be inhibited by HPT treatment.

## Materials and Methods

### Cell Culture

Rat chondrocytes were isolated and cultured as described in a previous study (Wang et al., 2019; Xu et al., 2019). Sprague Dawley (SD) rat knee cartilage tissues were cut into small pieces and digested with 2 mg/ml collagenase type II (Sigma, United States) for 4–6 h. The undigested tissues were removed, and the isolated cells were seeded in DMEM/F12 (1:1) medium (Gibco, United States) containing 10% fetal bovine serum (FBS) (Gibco, United States). Cells from the second or third passages were used in cell experiments. Animal experiments for this study were approved by the Institutional Review Board (IRB) of Chongqing University.

### Hesperetin and TNF-α Treatment

Hesperetin (HPT) and dexamethasone (DEX) were purchased from Selleck (Selleck Chemicals, United States) and then dissolved in DMSO as a 10 mM stock solution. Rat chondrocytes were treated with HPT at different concentrations (1, 5, 10, 20, 50, and 100 μM); DMSO was used as a control. Meanwhile, exposure to 10 μM HPT was implemented at different time intervals (1, 2, 3, 4, and 5 days). Morphology caused by HPT at different concentrations (1, 5, 10, 20, 50, and 100 μM) was detected by microscopy (Olympus, Japan). In addition, 10 ng/ml of TNF-α alone (the TNF-α-treated group) or TNF-α plus HPT at 10 μM (TNF-α + HPT-treated group) or TNF-α plus DEX at 10 μM (TNF-α + DEX-treated group) was used for treating chondrocytes for 48 h (TNF-α and HPT or TNF-α and DEX were added in the same order). All the experiments were performed in triplicate independently.

### Cell Viability Assay

Cell Counting Kit 8 (CCK-8) (Beyotime, China) was used to investigate cell viability and cytotoxicity. Chondrocytes were seeded at a density of 1,000 cells/well in a 96-well plate and tested for toxicity for 1–5 days with different concentrations of HPT. DMSO was used as a control. At designated time intervals, the cells were incubated with 10% CCK-8 reagent at 37°C for 2–4 h. After incubation, the corresponding absorbance was measured with a microplate reader at a wavelength of 490 nm. The data were analyzed with GraphPad 8.0. All the experiments were performed in triplicate independently.

### RT-qPCR Analysis

Chondrocytes were treated with TNF-α alone or TNF-α plus HPT and then harvested for the qRT-PCR assay. DMSO was used as control. Each sample was harvested using TRIzol reagent, and RNA was isolated. Total RNA was reverse transcribed into cDNA using the RevertAid First Strand cDNA Synthesis Kit (Thermo Fisher Scientific). Then, the cDNA was used as template to perform real-time polymerase chain reaction (PCR) on a StepOne Plus thermal cycler (Applied Biosystems). Primers were designed by NCBI primer blast and synthesized using Invitrogen. Data were analyzed according to the 2−ΔΔct method.

### Western Blotting

Rat chondrocytes were lysed by RIPA lysis buffer to extract the total protein which was separated by SDS-PAGE gel electrophoresis and transferred to PVDF (polyvinylidene fluoride) membrane (0.2 μm; BioRad). After 1 h blocking with 5% skimmed milk, the membrane was incubated with primary antibody ACAN (Abcam, ab36861), COL2A (Santa Cruz, sc-7763), IL-1β (Proteintech, 66,737-1-lg), IL-6 (Zen Bio, 347023), MMP-13 (Zen Bio, 511755), PTGS2 (Zen Bio, 383967), Bcl-2 (Zen Bio, 381702), CDK1 (Abcam,ab18), Cyclin D1 (Zen Bio, 382442), p-AMPKα1 (Zen Bio, 310044), AMPKα (Zen Bio, 380431), and GAPDH (Zen Bio, 384404) antibodies overnight at 4°C. After washing 3 times with 1×TBST, the membrane was incubated with horseradish peroxidase-labeled secondary antibody (goat anti-rabbit/mouse IgG, 1:5,000) at room temperature for 1 h. Finally, target protein was visualized by the ECL Western Detection Kit (Thermo Fisher Scientific, United States) and quantified by ImageJ software.

### Cell Cycle Flow Cytometric Analysis

Flow cytometry was used to determine the effect of HPT on the chondrocyte cycle. After being treated with TNF-α alone or TNF-α plus HPT, the chondrocytes were harvested and washed, followed by fixation using 70% alcohol at 4°C overnight. After washing the cells with PBS, they were treated with ribonuclease RNase A and incubated with propidium iodide (PI) for the cell cycle analysis on a BD FACSVerse^TM^ flow cytometer (BD Bioscience, San Jose, CA).

### RNA Sequencing

RNA sequencing was performed on chondrocytes in different treatments to determine the changes in their mRNA expression profiles. RNA extraction kit (Thermo Fisher Scientific, Waltham, MA) was used to extract total RNA from each group of cells. Send the obtained RNA sample to BGI Co., Ltd., Shenzhen, China, and perform RNA-seq operation on BGISEQ-500. The R language program was used to further analyze the sequencing results to find differential genes in the chip expression profile. Then, use the R program to perform the gene ontology (GO) biological function enrichment analysis and genome encyclopedia (KEGG) signal pathway enrichment analysis. All samples were repeated three times.

### Immunostaining Assays

The chondrocytes were seeded on coverslips coated with 0.01% poly L-lysine. After treatment, the cells were fixed in 4% paraformaldehyde for 30 min, followed by rinsing with PBS and permeating with 0.1% Triton X-100 on ice for 5 min. Then, the cells were blocked with a blocking solution at 37°C for 1 h and then incubated with the primary antibody p-AMPKα1 (Zen Bio, 310044) at 4°C overnight and with a fluorescently labeled secondary antibody (concentration 1:200) at 37°C for 1 h, respectively. After counterstaining with DAPI, the samples were imaged and captured under a fluorescence microscope (Olympus, Japan).

### Animal Model

The animal experiments were carried out following the protocol approved by the Animal Care and Use Committee of Chongqing University. The OA model was achieved by anterior cruciate ligament transection (ACLT) in SD rats, as described in our previous study (Wang et al., 2020a; Xu et al., 2021). There are 24 rats randomly assigned to four groups: healthy, OA (normal saline group), DEX, and HPT groups. Four weeks after ACLT surgery, 0.5 mg/kg DEX was injected into the knee joint in the DEX group, and 10 mM HPT was injected into the knee joint in the HPT group. Four weeks after drug treatment, all the rats were sacrificed by carbon dioxide anesthesia, then cervical dislocation, and articular cartilage samples were collected for microscopic evaluations. Six samples were collected in each group.

### Histological Analysis

The samples of the OA animal model were collected 8 weeks after ACLT. First, the knee joints of the rats were fixed in 4% paraformaldehyde for 2 days. Subsequently, they were decalcified in 10% EDTA solution for 2 months, dehydrated with a concentration gradient of alcohol, embedded in paraffin, and cut into 10 μm thick paraffin sections. All specimens were stained with hematoxylin and eosin (H&E) and safranin-O fast green. H&E staining was used to observe changes in the surface of articular cartilage, cartilage levels, and the number and morphology of cartilage cells. In addition, the histological immunofluorescent staining for p-AMPKα1 (Zen Bio, 310044) was performed to verify HPT exhibits the effect *in vivo* on regulating the AMPK pathway. In the microscopic examination, the histological score was scored according to the histological evaluation performed in the previous study (Xu et al., 2019; Wang et al., 2020b). The (SD) score of each group was the content of cartilage matrix captured from the safranin-O-positive staining area by ImageJ software. In short, we selected the ROI of the red area of the cartilage matrix in each group, and then, calculate the intensity value of the algorithm built into the ImageJ software for further statistical analysis. The statistical analysis is based on the mean ± SD.

### Statistical Analysis

SPSS 22.0 software package was used to perform statistical processing. One-way analysis of variance was used to determine significant differences between groups. The experimental data were expressed as mean ± SD (mean ± SEM), and *p* < 0.05 was considered statistically significant.

## Results

### Cytotoxicity of HPT to Chondrocytes

**FIGURE 1:**
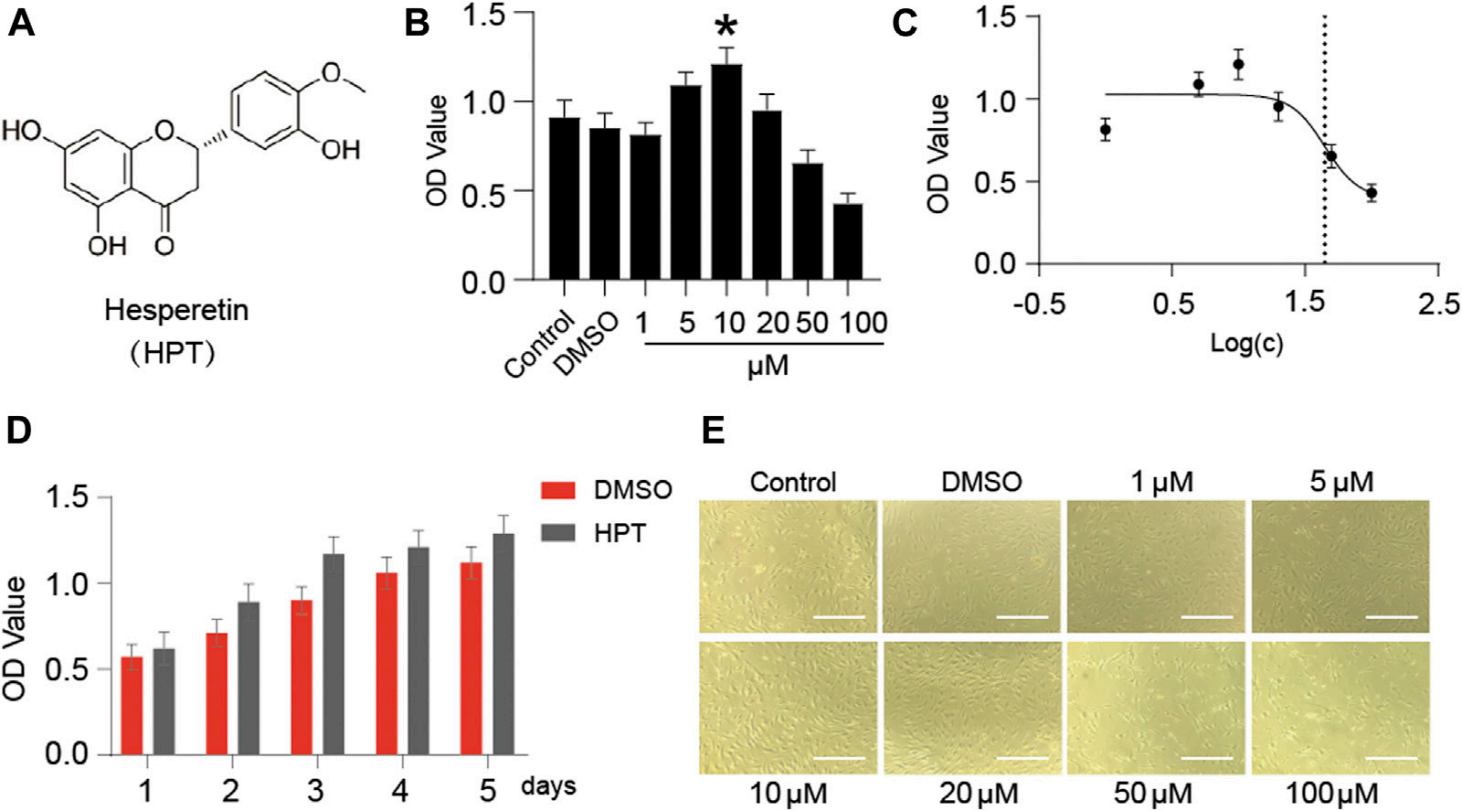
Cytotoxicity of HPT-treated chondrocytes. **(A)** The molecular structure of HPT. **(B)** Different concentrations (1, 5, 10, 20, 50, and 100 μM) of HPT were cultured for 48 h, with *p < 0.05 as a significant difference. **(C)** The IC50 of HPT to chondrocytes, and the concentration is transferred to Log(c). **(D)** HPT treated 10 µM and controlled the cell proliferation curve for 5 days. **(E)** Photographs of cells cultured with different concentrations (1, 5, 10, 20, 50, and 100 μM) of HPT for 48 h.

Figure 1A presents the chemical structure of HPT. CCK-8 assay was used to investigate the cytotoxic effects of HPT on SD rat chondrocytes. The results showed no apparent cytotoxic effects of HPT on chondrocytes at concentrations of 0–20 μM; however, it had cytotoxic effects at concentrations ≥50 μM (Figure 1B). In addition, HPT promoted the proliferation of chondrocytes slightly at 10 μM concentration. Therefore, 10 μM HPT was the concentration selected for further studies. We further investigated the cell proliferation rate after treatment with 10 μM HPT for 5 days. Compared with the control group (DMSO), supplementation with 10 μM HPT increased cell proliferation (Figure 1D). There was no change in the morphology of chondrocytes after treatment with 1–20 μM HPT; however, chondrocytes exposed to 50 and 100 μM HPT showed significant morphological changes, and cell counts decreased in a dose-dependent pattern as well (Figure 1E).

### HPT Recoveries TNF-α-Induced Abnormal Gene and Protein Expression

**FIGURE 2:**
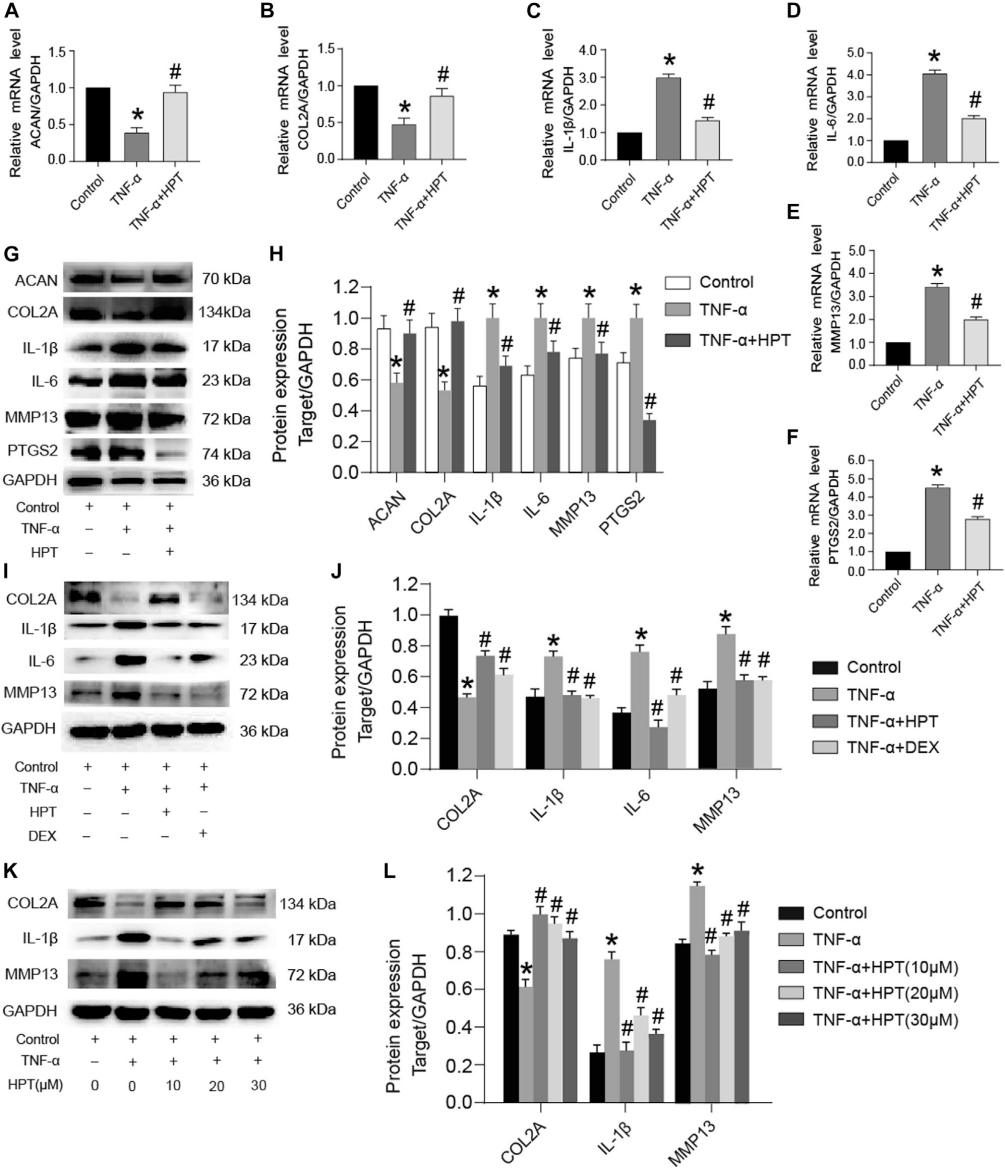
HPT recovered TNF-α-induced abnormal expression of genes and proteins. **(A–F)** After stimulated chondrocytes with TNF-α and then treated them with or without 10 μM HPT for 48 h, the mRNA expression levels of ACAN, COL2A, IL-1β, IL-6, MMP-13, and PTGS2. **(G, H)** After stimulated chondrocytes with TNF-α and then treated them with or without 10 μM HPT for 48 h, the protein expression levels of IL-1β, IL-6, PTGS2 and (cartilage matrix markers) COL2A, ACAN, MMP13. **(I, J)** After stimulated chondrocytes with TNF-α and then treated them with/without 10 μM HPT or DEX for 48 h, the protein expression levels of IL-1β, IL-6, and (cartilage matrix markers) COL2A and MMP13. **(K, L)** After stimulated chondrocytes with TNF-α and then treated them with or without 10, 20, and 30 μM HPT for 48 h, the protein expression levels of IL-1β and (cartilage matrix markers) COL2A and MMP13. According to the intensity of semiquantitative IOD through the ImageJ software, *p < 0.05 was considered as a significant difference from the control group; #*p* < 0.05 was considered as a significant difference from TNF-α.

Chondrocytes are generally very sensitive to TNF-α, which reduces the aggrecan and collagen type II synthesis and promotes the development of OA. To investigate the protective function of HPT in TNF-α-treated chondrocytes, we examined the effect of HPT on aggrecan and collagen type II using the Western blotting analysis and RT-PCR. Moreover, we then examined the impact of HPT on TNF-α-induced production of IL-1β, IL-6, MMP13, and PTGS2 in chondrocytes. The cells were treated with 10 μM HPT and 10 ng/ml of TNF-α for 48 h. Compared with the control group, TNF-α significantly increased mRNA levels of IL-1β, IL-6, MMP-13, and PTGS2 and inhibited ACAN and COL2A gene expression (*p < 0.05; TNF-α vs. control), while HPT recovered these gene levels (#*p* < 0.05; TNF-α + HPT vs. TNF-α) (Figures 2A–F). Like gene expression results, HPT restored TNF-α-induced downregulation of COL2A and ACAN and reversed TNF-α-induced IL-1β, IL-6, MMP-13, and PTGS2 protein production (Figures 2G,H). Moreover, DEX also restored TNF-α-induced downregulation of COL2A and reversed TNF-α-induced IL-1β, IL-6, and MMP-13 protein production (Figures 2I,J). Finally, 20 and 30 μM HPT also restored TNF-α-induced downregulation of COL2A and reversed TNF-α-induced IL-1β and MMP-13 protein production (Figures 2K,L). These findings indicated that HPT could activate cartilaginous ECM synthesis and suppress the associated TNF-α-induced expression of inflammatory genes.

### Effects of HPT on TNF-α-Induced Expression of Proliferative Gene/Protein

**FIGURE 3:**
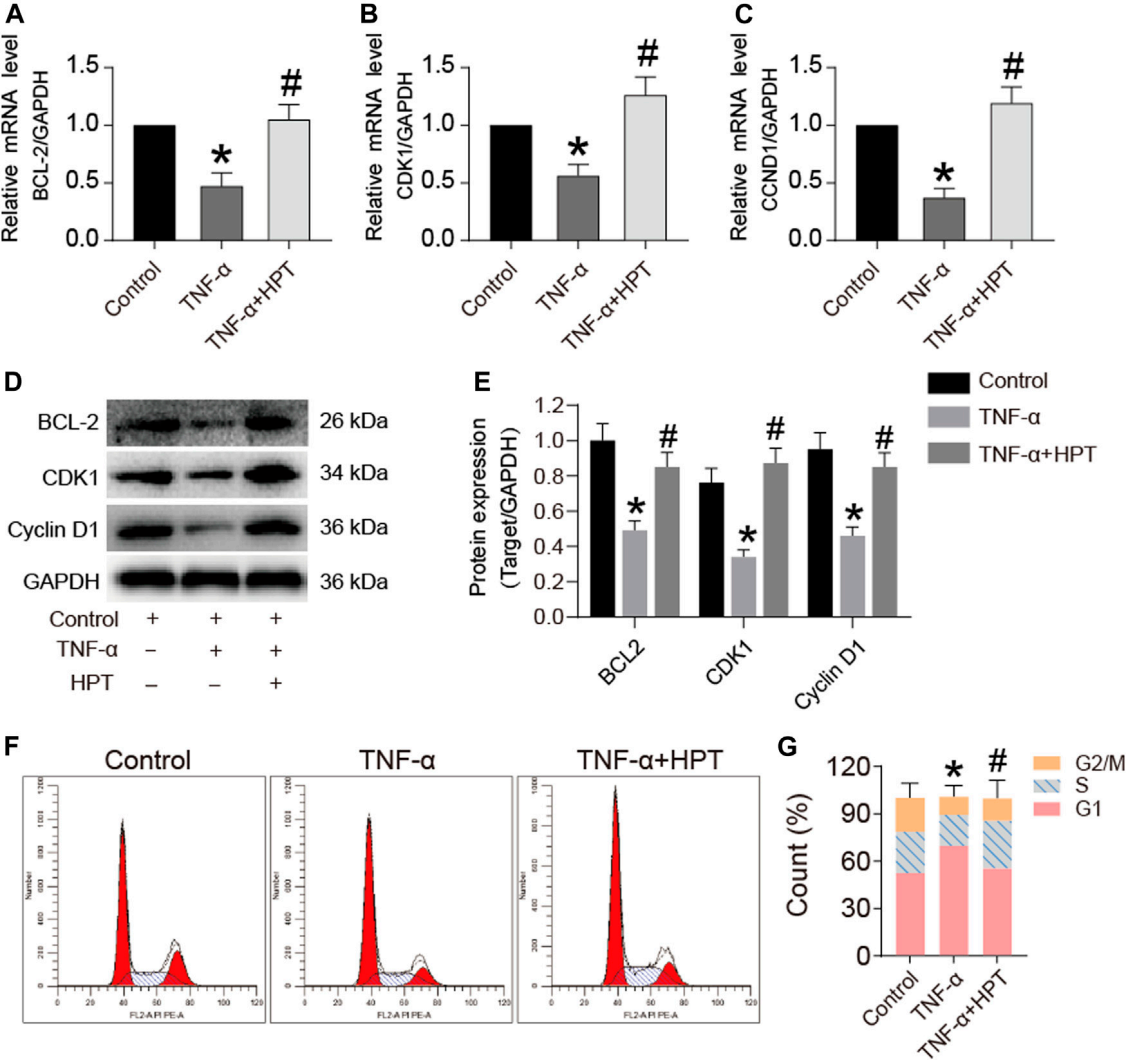
The effect of HPT on the cell cycle of chondrocytes induced by TNF-α. **(A–E)** The expression levels of apoptosis and cycle-related genes and proteins after stimulated chondrocytes with TNF-α and then treated them with or without 10 μM HPT for 48 h. *P < 0.05 was considered as a significant difference from the control group; #*p* < 0.05 was considered as a significant difference from TNF-α. **(F, G)** Flow cytometry used to detect the effect of HPT on the chondrocytes cycle. Counted the percentages of the G2/M phase, s phase, and G1 phase, make statistical comparisons. There are three replicates for each concentration point. *P < 0.05 was considered as a significant difference from the control group; #*p* < 0.05 was considered as a significant difference from TNF-α.

The qRT-PCR and Western blotting were used to investigate the gene and protein expression of Bcl-2, CDK1, and Cyclin D1. The results showed that TNF-α significantly decreased Bcl-2, CDK1, and Cyclin D1 mRNA levels (*p < 0.05; TNF-α vs. control), while HPT recovered these gene levels (#*p* < 0.05; TNF-α + HPT vs. TNF-α) (Figures 3A–C). Similar to the transcription level, the decreased Bcl-2, CDK1, and Cyclin D1 levels induced by TNF-α were restored to the control group level in the group with TNF-α + HPT (*p < 0.05, TNF-α vs. control; #*p* < 0.05, TNF-α + HPT vs. TNF-α) (Figures 3D,E). We further investigated the cell cycle to determine whether HPT protects chondrocytes against the inhibitory effect of TNF-α on the proliferation of chondrocytes by flow cytometry. The results showed significant decreases in the S phase following TNF-α treatment (*p < 0.05; TNF-α vs. control), while the percentages of chondrocytes in the S phase were restored to the control group level in the group with TNF-α + HPT (#*p* < 0.05; TNF-α + HPT vs. TNF-α) (Figures 3F,G).

### DEGs Identification and Pathway Enrichment Analysis

**FIGURE 4:**
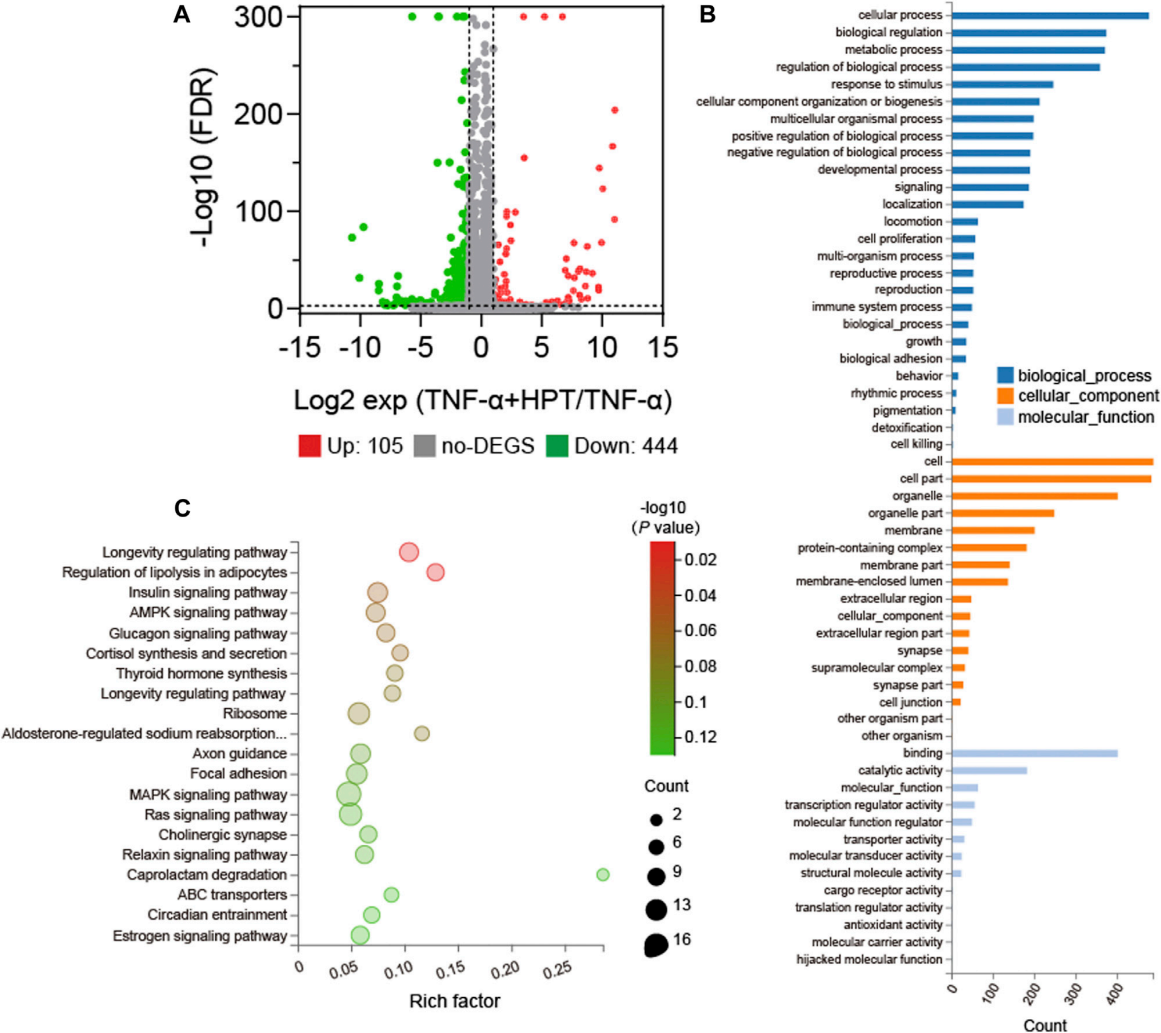
Gene expression profile with or without 10 µM HPT treatment. **(A)** Volcano map of differentially expressed genes (DEGs) in HPT and control (upregulation: 105; downregulation: 444), FC (fold change) > 1 was considered as a positive DEGs. **(B)** GO enrichment those common choices for DEGs including biological processes (blue), cellular components (orange), and molecular functions (light blue). **(C)** Enrichment bubble chart based on the KEGG enrichment analysis. A larger *p* value indicates a higher degree of enrichment.

The RNA-seq experiments were performed in cells affected by TNF-α induction with or without HPT treatment to further investigate the protective and anti-inflammatory mechanism of HPT in TNF-α-induced chondrocytes. The results demonstrated that compared with the TNF-α group, there were many differentially expressed genes (DEGs) in chondrocytes treated with TNF-α and 10 μM HPT, of which 105 genes were upregulated and 444 genes were downregulated (Figure 4A). Furthermore, GO functional annotations were made on the identified DEGs, which were highly involved in cell processing, biological regulation, metabolic processes, and biological process regulation (Figure 4B). Furthermore, the KEGG signaling pathway enrichment analysis indicated that these DEGs were highly enriched in functions related to the longevity-regulating pathway, regulation of lipolysis in adipocytes, insulin signaling pathway, AMPK signaling pathway, and glucagon signaling pathway (Figure 4C).

### HPT Relieves TNF-α-Induced Inflammation of Chondrocytes through the AMPK Signaling Pathway

**FIGURE 5:**
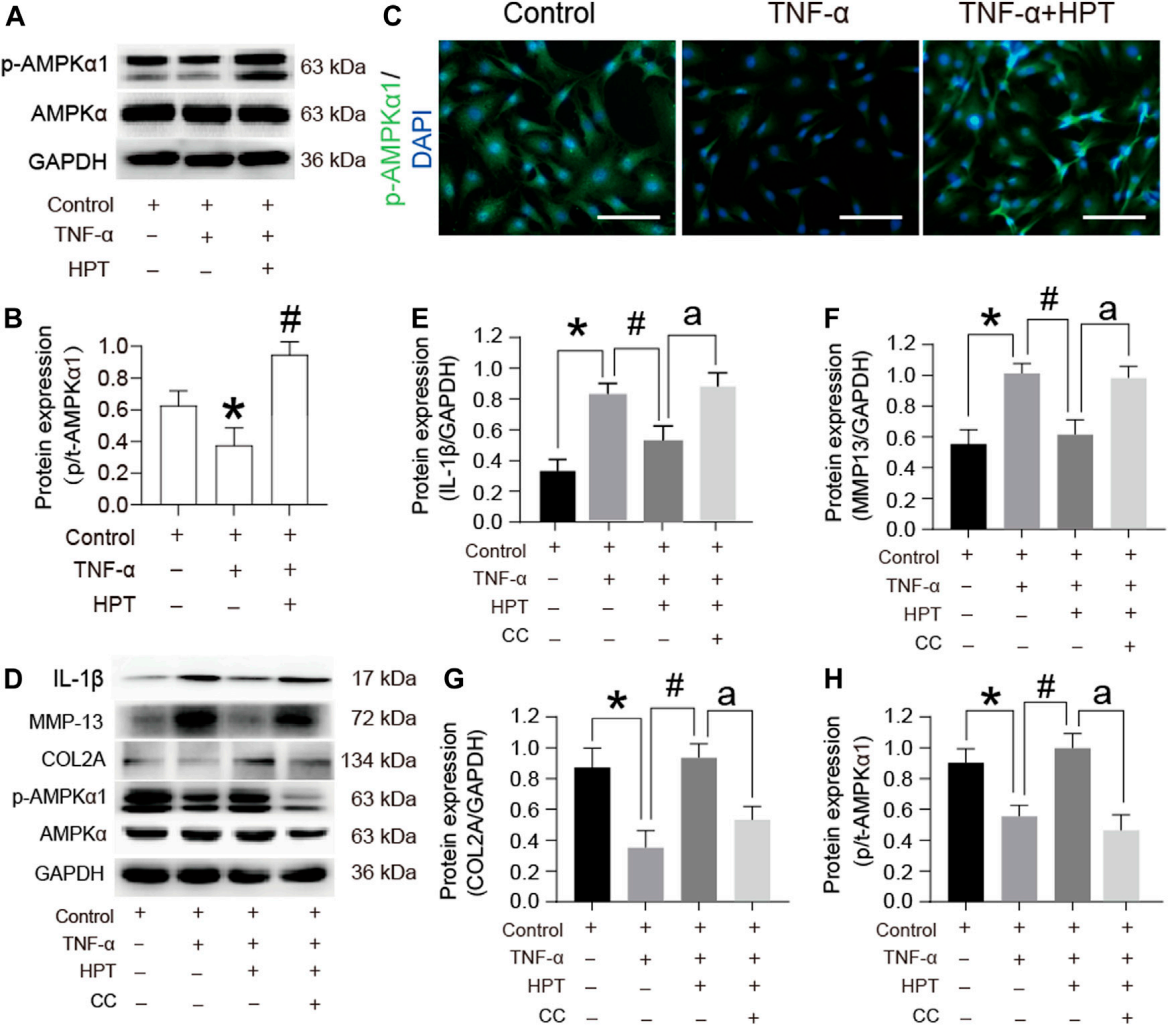
AMPK signaling pathway participates in the protective effect of HPT on TNF-α-stimulated rat chondrocytes. **(A, B)** Protein expression profile of p-AMPKα1 cultured under TNF-α conditions with or without HPT. **(C)** Immunofluorescence of p-AMPKα1 in chondrocytes treated with TNF-α and/or HPT. **(D–H)** Protein expression profile of IL-1β, MMP13, COL2A, and p-AMPKα1 cultured under the condition of HPT with or without and TNF-α with or without CC. For statistical analysis, *p < 0.05 was considered as a significant difference from the control group; #*p* < 0.05 was considered as a significant difference from TNF-α. ^a^ *P* < 0.05 was considered as a significant difference from TNF-α + HPT.

Based on the results of highly enriched signaling pathways, we selected AMPK signaling pathways for further analyses. Western blot analysis showed that TNF-α induction suppressed the phosphorylated-/total-AMPKα1 (p/t-AMPKα1) ratios; however, the phosphorylation of AMPKα1 increased after HPT supplementation (Figures 5A,B) (*p < 0.05). Furthermore, immunofluorescence staining with p-AMPKα1 was performed, which further supported the Western blot results (Figure 5C), indicating that HPT plays a key role in the activation of AMPK phosphorylation in chondrocytes. To determine the role of the AMPK signaling pathway in HPT-induced alleviation of inflammation, we examined the effect of HPT on TNF-α-induced inflammation with or without compound C, a selective AMPK inhibitor. Inhibition of AMPK activation by compound C abolished the HPT-induced decrease in IL-1β and MMP-13 levels (Figures 5D–F) (^a^ *p* < 0.05). In addition, it also diminished the upregulation of p-AMPKα1 and COL2A expression by HPT treatment (Figures 5D,G,H) (^a^ *p* < 0.05). These data suggested that HPT relieves TNF-α-induced inflammation of chondrocytes through the AMPK signaling pathway.

### HPT Prevents Cartilage Degradation in the Rat Model

**FIGURE 6:**
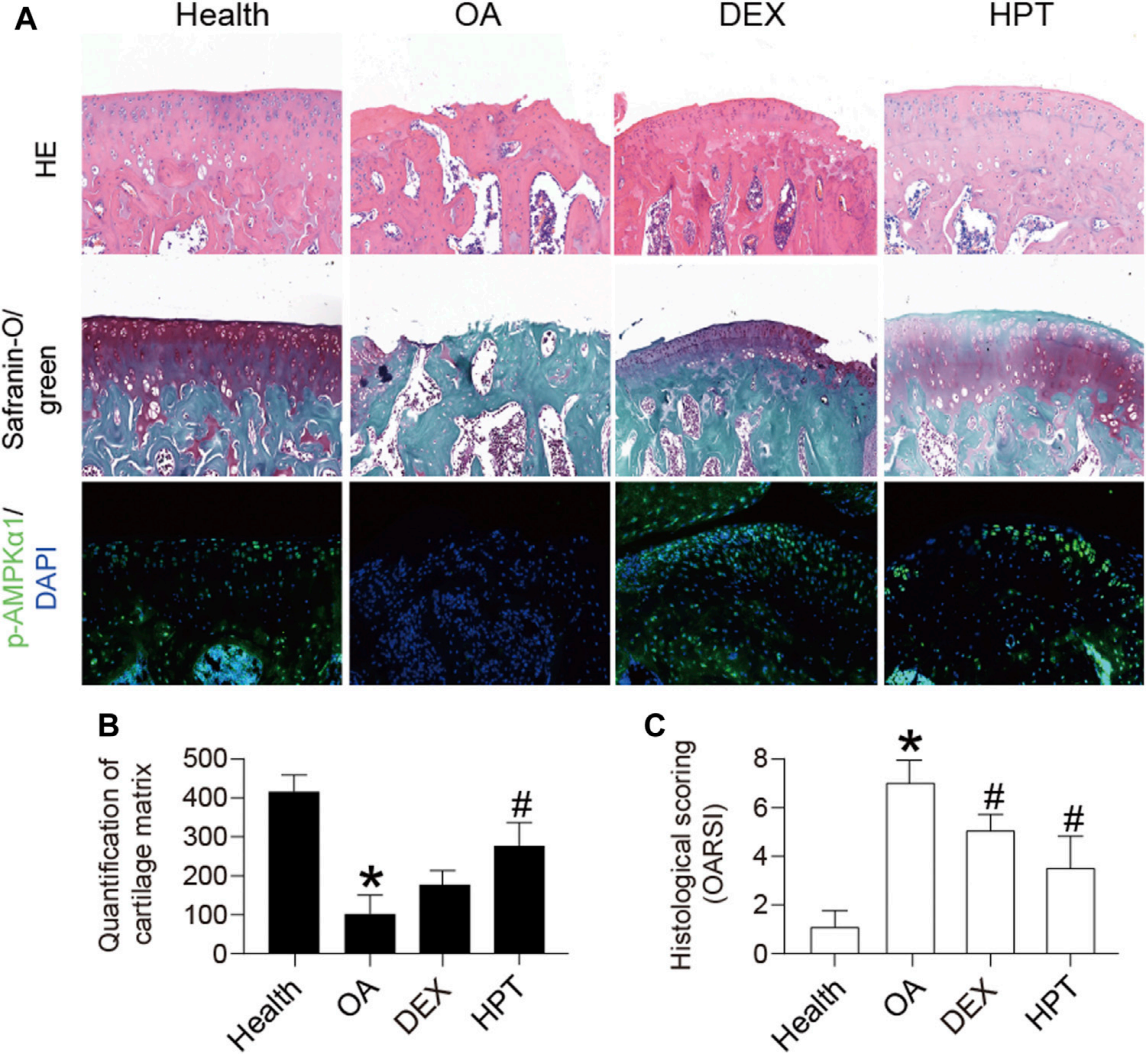
Histological analysis of knee cartilage after HPT treatment (H&E, safranin-O/green, and IF), magnified 100 times. For statistical analysis, *p < 0.05 was considered statistically significant compared with healthy; #*p* < 0.05 was considered statistically significant with OA. DEX, dexamethasone as positive control.

ACLT was used to establish the rat OA model. Healthy rats served as a control group. HPT was locally injected into the joint cavity 4 weeks after ACLT surgery. As shown in Figure 6, the articular cartilage had a regular shape in the healthy group. The articular cartilage morphology in the OA group was uneven, with reduced cartilage thickness compared with the healthy group. The articular cartilage in the HPT group was more regular, with significantly higher cartilage thickness compared with the OA group. The cartilage matrix of the OA group was significantly thinner than that of the healthy group (*p < 0.05). HPT treatment significantly changed this situation (#*p* < 0.05). In addition, compared with the healthy group, the histological score of the OA group was significantly different (*p < 0.05); however, DEX and HPT treatment significantly improved cartilage degeneration, and the histological score was significantly different from that of the OA group (#*p* < 0.05), indicating that HPT treatment successfully inhibited cartilage degeneration. In addition, the expression of p-AMPKα1 was measured *in vivo* by immunofluorescent staining (Figure 6). The result showed that TNF-α induction suppressed the phosphorylation of AMPKα1; however, the phosphorylation level of AMPK*α*1 was recovered after DEX and HPT supplementation.

## Discussion

OA is a chronic disease with low-grade inflammation (Haseeb and Haqqi, 2013; Bartels et al., 2016). Thus, inhibition of inflammation is considered a strategy to delay the progression of OA (Robinson et al., 2016). However, there is currently no medication available to cure OA or decelerate its progression. Recently, an increasing number of natural compounds have caused concerns as ideal drugs for OA due to their anti-inflammatory activities and limited side effects (Wang et al., 2020a). Thus, a drug, like HPT, with an anti-inflammatory function that delays the structural progression of the disease, represents a novel class of drugs to treat OA.

Growing evidence indicates that HPT positively affects anti-inflammatory reactions (Spagnuolo et al., 2018; Khan et al., 2020). Inflammatory mediators, such as TNF-α, IL-1β, and IL-6, can induce metabolic factors such as PTGS2 and MMPs, leading to articular cartilage degeneration (Oliviero et al., 2020). It has been reported that HPT exerts marked anti-inflammatory effects in mice with neurodegeneration (Ikram et al., 2019) and postmyocardial infarction (Wang et al., 2017). It has also been reported that HPT can significantly inhibit IL-1β-induced inflammatory response in human chondrocytes in osteoarthritis (Lin et al., 2020). In this study, HPT activated cartilaginous ECM synthesis and suppressed the associated TNF-α-induced expression of inflammatory genes. Furthermore, HPT at 10 μM concentration promoted the proliferation of chondrocytes slightly. The RT-qPCR, Western blotting, and flow cytometry results also showed that HPT reversed the inhibitory effect of TNF-α on chondrocyte proliferation. It also has been reported that HPT can protect HK-2 cells by reducing the apoptosis induced by cisplatin (Chen et al., 2019). The above finding shows that HPT can protect chondrocytes. However, the related signaling pathways and molecular mechanisms of HPT in chondrocytes remain unclear.

HPT can inhibit inflammatory responses in lipopolysaccharide-induced RAW 264.7 cells via the inhibition of the NF-κB pathway and activation of the Nrf2/HO-1 pathway (Ren et al., 2016). Researchers found that HPT inhibits IL-1β-induced inflammation in human OA chondrocytes by suppressing NF-κB and initiating the Nrf2/HO-1 pathway (Lin et al., 2020). However, the RNA-seq experiment was used to investigate the protective and anti-inflammatory mechanisms of HPT in TNF-α-induced chondrocytes in the present study. Enrichment analysis of the KEGG signaling pathway showed that important DEGs are highly enriched in the longevity regulating pathway, regulation of lipolysis in adipocytes, insulin signaling pathway, AMPK signaling pathway, and glucagon signaling pathway, among which activating the AMPK signaling pathway can slow down the development and progression of OA (Li et al., 2020). AMPK is the main regulator of energy balance and metabolism. Decreased AMPK activity can be assessed by AMPK-catalyzed phosphorylation of a specific threonine in the α subunit (AMPKα1) (Stein et al., 2000). Therefore, we evaluated the phosphorylation level of AMPKα1 in chondrocytes and showed that TNF-α reduced the phosphorylation level of AMPKα1. Immunofluorescence staining also confirmed this finding, which is consistent with previous studies demonstrating that decreased phosphorylation of AMPKα1 is also seen in chondrocytes in response to TNF-α (Terkeltaub et al., 2011). Furthermore, flavonoids have always been considered natural AMPK agonists, and many of their pharmacological activities depend on the activation of AMPK (Gentile et al., 2018). The present study showed that HPT plays a key role in the activation of AMPK phosphorylation in chondrocytes.

We examined the effect of HPT on TNF-α-induced inflammation with or without compound C, a selective AMPK inhibitor, to determine the role of the AMPK signaling pathway in HPT-induced alleviation of inflammation. Further verification experiments showed that HPT relieves TNF-α-induced inflammation of chondrocytes through the AMPK signaling pathway. It has also been demonstrated that AMPKα1 deficiency in chondrocytes accelerates the development of both injury-induced and age-related spontaneous OA in mice (Zhou et al., 2017). Furthermore, it is well known that the activation of AMPK can inhibit the inflammatory response by inhibiting the function of the NF-κB signaling pathway (Salminen et al., 2011). Therefore, we speculate that HPT can be regulated through the AMPK signaling pathway to protect chondrocytes.

This study established an ACLT rat model and locally administered HPT treatment to further determine the therapeutic effect of HPT on OA. The ACLT-induced OA model we established is very similar to human osteoarthritis, and our previous studies have been reported (Wang et al., 2020a). It has also been reported that the ACLT model accompanied the activation of TNF-α (Xie et al., 2018). Besides, restabilization of joint instability by ACLT may suppress inflammatory cytokines (TNF-α), thereby delaying the progression of OA (Murata et al., 2017). Previous studies have also demonstrated that HPT can inhibit the development of OA induced by the DMM model (Lin et al., 2020). Our research results showed that HPT treatment could significantly inhibit the inflammatory response of the ACLT-induced OA model. Furthermore, HPT treatment could delay the progression of articular cartilage degeneration, and the cartilage defects in the rat knee joint were significantly restored, with increased cartilage thickness. Furthermore, the *in vivo* study provided evidence that HPT prevented the occurrence and development of OA by reducing cartilage degradation, delaying cartilage surface erosion, and maintaining chondrocyte formation. In conclusion, this study suggests that HPT might be used as a potential therapeutic agent for OA.

## Data Availability

The datasets presented in this study can be found in online repositories. The names of the repository/repositories and accession number(s) can be found in the following: NCBI BioProject PRJNA755303.
